# Preservation and Taphonomy of Fossil Insects from the Earliest Eocene of Denmark

**DOI:** 10.3390/biology11030395

**Published:** 2022-03-03

**Authors:** Miriam Heingård, Peter Sjövall, Bo P. Schultz, René L. Sylvestersen, Johan Lindgren

**Affiliations:** 1 Department of Geology, Lund University, SE-223 62 Lund, Sweden; johan.lindgren@geol.lu.se; 2 Materials and Production, RISE Research Institutes of Sweden, SE-501 15 Borås, Sweden; peter.sjovall@ri.se; 3 Fur Museum, Museum Salling, DK-7884 Nederby, Denmark; bosc@museumsalling.dk (B.P.S.); rlsy@museumsalling.dk (R.L.S.)

**Keywords:** cuticle, Eocene, Fur Formation, insects, melanin, mo-clay, pigment, Stolleklint Clay, structural coloration, Ølst Formation

## Abstract

**Simple Summary:**

Insect fossils dating 55 million-years-old from the Stolleklint Clay and Fur Formation of Denmark are known to preserve both fine morphological details and color patterns. To enhance our understanding on how such fragile animals are retained in the fossil record, we examined a pair of beetle elytra, a wasp and a damselfly using sensitive analytical techniques. In our paper, we demonstrate that all three insect fossils are composed of cuticular remains (that is, traces of the exoskeleton) that, in turn, are dominated by the natural pigment eumelanin. In addition, the beetle elytra show evidence of a delicate lamellar structure comparable to multilayered reflectors that produce metallic hues in modern insects. Our results contribute to improved knowledge on the process of fossilization of insect body fossils in marine environments.

**Abstract:**

Marine sediments of the lowermost Eocene Stolleklint Clay and Fur Formation of north-western Denmark have yielded abundant well-preserved insects. However, despite a long history of research, in-depth information pertaining to preservational modes and taphonomic pathways of these exceptional animal fossils remains scarce. In this paper, we use a combination of scanning electron microscopy coupled with energy-dispersive X-ray spectroscopy (SEM-EDX), transmission electron microscopy (TEM) and time-of-flight secondary ion mass spectrometry (ToF-SIMS) to assess the ultrastructural and molecular composition of three insect fossils: a wasp (Hymenoptera), a damselfly (Odonata) and a pair of beetle elytra (Coleoptera). Our analyses show that all specimens are preserved as organic remnants that originate from the exoskeleton, with the elytra displaying a greater level of morphological fidelity than the other fossils. TEM analysis of the elytra revealed minute features, including a multilayered epicuticle comparable to those nanostructures that generate metallic colors in modern insects. Additionally, ToF-SIMS analyses provided spectral evidence for chemical residues of the pigment eumelanin as part of the cuticular remains. To the best of our knowledge, this is the first occasion where both structural colors and chemical traces of an endogenous pigment have been documented in a single fossil specimen. Overall, our results provide novel insights into the nature of insect body fossils and additionally shed light on exceptionally preserved terrestrial insect faunas found in marine paleoenvironments.

## 1. Introduction

Lowermost Eocene deposits of the Limfjord Region, Northwestern Jutland, Denmark, have yielded diverse biotas of exceptionally preserved plant and animal body fossils that frequently retain soft parts, such as feathers and skin [1,2]. The local stratigraphic succession comprises the Fur Formation, a *Konservat-Lagerstätte*, and the underlying, less well known Stolleklint Clay of the Ølst Formation, which together constitute the so-called “mo-clay deposits” [3]. Despite being interpreted as representing a relatively deep marine offshore setting [4], the fine-grained sediments house a wealth of terrestrially derived organisms, of which insects are particularly conspicuous with more than 200 species described to date [1,2]. The insect fossils often preserve fine anatomical details, including segmentation, appendages, wings with well-defined venation, traces of original color patterns and sometimes even residual endogenous biomolecules [1,5].

Insects are currently one of the most ubiquitous and numerically abundant groups of animals on Earth, and they have a fossil record that dates back to the Early Devonian [6,7]. Exceptionally preserved biotas, such as those from the Limfjord Region, act as important “windows” into the evolutionary history of this clade, but are also crucial for understanding taphonomic pathways that may contribute to the retention of delicate anatomical features in the rock record. Recent research has made considerable progress with respect to insect fossilization processes (e.g., [8,9,10,11,12,13,14,15,16,17,18,19]). However, although previous work on insects from the Stolleklint Clay and Fur Formation has touched upon taphonomic and biostratinomic processes [20,21,22,23], most studies have focused on other aspects of the assemblage (e.g., [24,25,26,27,28]). Moreover, in-depth chemical and ultrastructural analyses that can provide valuable information on fossil preservation patterns have so far been conducted almost exclusively on vertebrate remains [29,30,31,32,33,34], while insects, despite their great abundance, merely have been the subject of a single investigation [5]. 

In the present contribution, we expand current knowledge on organic preservation by employing an integrated experimental approach to a selection of insect fossils from the Eocene of Denmark. We investigate and illustrate these specimens with the aim of achieving a better understanding of the biostratinomic, taphonomic and diagenetic processes that result in exceptional preservation. 

## 2. Geological Setting 

The fossils analysed in this study originate from the Ølst and Fur formations in the Limfjord Region, Northwestern Jutland, Denmark. In this area, the Ølst Formation is represented solely by the Stolleklint Clay—the lowermost unit of the formation [35,36]—which is directly overlain by the Fur Formation. The Stolleklint Clay consists of laminated, clays, whereas the Fur Formation comprises an approximately 60-m-thick sequence of clayey diatomite [37]. The clay sequence formed in a semi-restricted marine basin, well below the wave base under anoxic to dysoxic bottom conditions [35]. The diatomite facies of the Fur Formation have been interpreted as deriving from periodic diatom blooms associated with local upwelling [4,37,38,39]. Oxygen-depleted bottom conditions are indicated by the generally undisturbed bedding planes, an absence of a benthic biota, and well-preserved, often fully articulated fossils [37]. Both formations contain volcanic ash that originates from eruptions associated with the opening of the North Atlantic Ocean [40]. These layers are numbered in relation to an easily recognizable ash bed (denominated “+1”) that occurs in the middle part of the Fur Formation [41]. As of today, almost 200 volcanic ash layers have been recognized in the strata [37]. The processes of dating two of these layers (−17 and +19) have yielded ages of ~55.6 and ~55.4 Ma, respectively [42,43,44], placing both rock units in the earliest Eocene (Ypresian), during and immediately after the Paleocene-Eocene Thermal Maximum [45,46]. Calcareous concretions are common in the Fur Formation within certain horizons [37]. X-ray diffraction has shown that these consist of low Mg-calcite [47]. Carbon and oxygen isotope compositions further indicate that most of the carbonate has a bacterial origin, being formed as a result of metabolization of organic matter [47]. 

## 3. Material and Methods

### 3.1. Fossil Material

Three insect fossils from the Stolleklint Clay and Fur Formation showing various states of preservation were selected for this study: (1) a pair of isolated but three-dimensional beetle elytra (Coleoptera; FUM-N-17627) collected from a calcium carbonate concretion near ash layer +15 in the Fur Formation; (2) a flattened yet fully articulated wasp (Hymenoptera, Ichneumonidae; FUM-N-11263) preserved in a calcium carbonate concretion, collected from the Fur Formation on the Island of Mors; and (3) a compressed but largely articulated damselfly (Odonata, Zygoptera; FUM-N-10904) found in hardened clays of the Stolleklint Clay. All specimens are housed in collections at Museum Salling, Fur Museum, Fur, Denmark, and were photographed in 96% ethanol using an Olympus SZX16 stereo microscope equipped with an Olympus SC30 digital camera prior to ultrastructural and molecular analyses. 

### 3.2. Scanning Electron Microscopy and Elemental Analysis

All fossils were examined in a Zeiss Supra 40VP FEG-SEM using either an Everhart-Thornley type secondary electron detector (SE2) at an electron energy of 2 keV or a variable pressure secondary electron detector (VPSE) at 15 keV. Elemental analyses and mappings used a X-Max 50 mm^2^ silicon drift detector from Oxford Instruments at an electron energy of 15 keV. Complementary imaging and elemental analyses were conducted in a Tescan Mira3 High Resolution Schottky FEG-SEM linked to an energy-dispersive spectrometer (X-MaxN 80, 124 eV, 80 mm^2^) from Oxford Instruments using an electron energy of 15 keV. Samples obtained from the beetle elytra (FUM-N-17627) were coated with a 15nanometer-thick gold-palladium film prior to analysis, whereas FUM-N-11263 and FUM-N-10904 were examined uncoated. 

### 3.3. Transmission Electron Microscopy

TEM analyses were performed only on samples from the beetle elytra (FUM-N-17627) because the relict cuticle in FUM-N-11263 and FUM-N-10904 was too thin and spatially incoherent to allow meaningful sampling. Small pieces (~1 mm^2^) of elytra were removed from FUM-N-17627 using a sterile scalpel and then dehydrated in a graded ethanol series. Following this procedure, the samples were embedded in epoxy resin (Agar 100) via treatment with acetone. Ultra-thin sections (70 nm) were cut using a Leica EM UC7 ultramicrotome equipped with a diamond knife. The sections were then mounted on copper grids without additional treatment or staining and examined in a JEOL JEM-1400 Plus transmission electron microscope at 100 kV. 

### 3.4. Time-of-Flight Secondary Ion Mass Spectrometry

ToF-SIMS was used for molecular characterization of all fossils. In ToF-SIMS, the sample surface is bombarded by a focused beam of high energy ions, and molecular information is obtained from mass spectra acquired by the secondary ions emitted during this collision process [48]. By scanning the primary ion beam and acquiring mass spectra from each pixel in a selected analysis area, spatially resolved mass spectrometric data can be acquired, which in turn can be presented either as ion images (showing the signal intensity of selected secondary ions across the analysis area) or as mass spectra from selected regions of interest (ROIs) within the analysis area.

ToF-SIMS analyses were carried out in a TOFSIMSIV instrument (IONTOF GmbH, Münster, Germany) using 25 keV Bi_3_^+^ primary ions and low-energy electron flooding for charge compensation. Positive and negative ion data were acquired with the instrument optimized for either high mass resolution (bunched mode, m/Δm ≈ 3000, lateral resolution 3–5 µm) or high lateral resolution (m/Δm ≈ 300, lateral resolution 0.5–1 µm). Spectra and images were generated using the SurfaceLab software (version 7.1, IONTOF GmbH). 

Principal components analysis (PCA) of mass spectral data was conducted using the Solo software (version 7.9.5, Eigenvector Research, Inc., Manson, WA, USA), employing Poisson scaling and prior normalization of the peak intensities to the sum intensity of all included peaks. The analysis included all major “eumelanin” peaks (43 in total) in the mass range *m/z* 48–146 (see [49]). Reference spectra were acquired from pure calcium carbonate (Sigma-Aldrich Sweden AB, Stockholm, Sweden), eumelanin (from *Sepia officinalis*; Sigma-Aldrich Sweden AB) and synthetic eumelanin (Fisher Scientific GTF AB, Göteborg, Sweden). 

## 4. Results

### 4.1. FUM-N-17627

**Figure 1:**
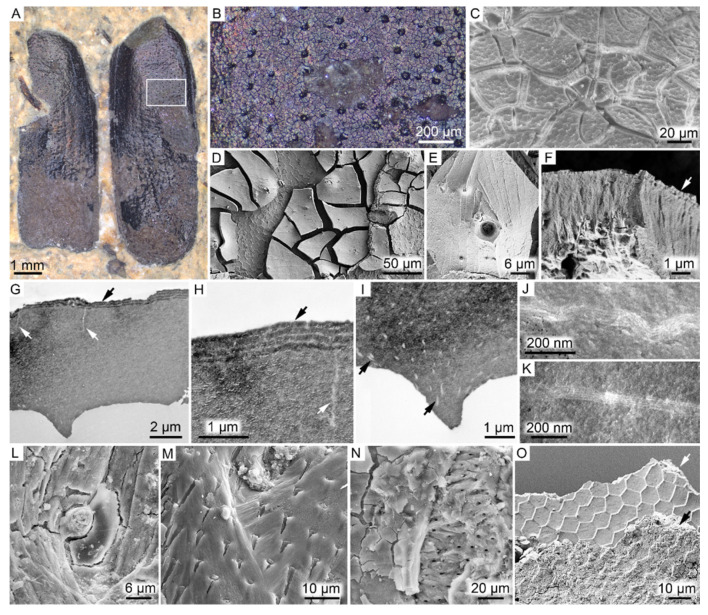
FUM-N-17627 (Coleoptera). (**A**) The fossil elytra in dorsal view prior to sampling, displaying a faint metallic shine under conventional light. (**B**) Higher magnification of the dark matter from the area demarcated in (**A**). (**C**) SEM micrograph of the external (outer) surface of the elytra. Note hexagonal patterning and desiccation cracks. (**D**,**E**) SEM micrographs of the smooth internal surface of the elytra. Note hexagonally arranged imprints in the sedimentary matrix neighboring the fossil matter. (**F**) SEM micrograph showing a section through the predominantly amorphous cuticular matrix. Note thin layers along the external margin of the elytra (indicated by an arrow). (**G**) TEM micrograph depicting a vertical section through the elytra. Note largely amorphous interior save for thin subvertical rifts (white arrows) and four electron-dense layers that alternate with thinner, electron-translucent bands (black arrow). (**H**) Higher magnification of the thin structures (white arrow) and epicuticular layering (black arrow). (**I**) TEM micrograph highlighting undulating, electron-lucent fibrillar nanostructures (arrows) in the otherwise largely homogenous cuticular matrix. (**J**,**K**) Higher magnification of the structures depicted in (**I**) showing individual filaments. (**L**–**N**) SEM micrographs depicting remains of putative seta, sensilla or microtrichia-like features. (**O**) SEM micrograph showing the two mineral phases identified in direct association with the elytra. The fine texturing with distinct polygonal imprints of the calcium-rich phase (white arrow) is clearly distinguishable from the more coarse-grained iron-rich and silicon-rich phase (black arrow).

FUM-N-17627 comprises a pair of three-dimensionally preserved beetle elytra with a faint metallic shine when visualized under conventional light (Figure 1A). At higher magnification, the elytra consist of fragmented, dark-colored matter that is regularly perforated by distinct pits (Figure 1B). SEM analysis further showed that the external surface of the fragments has a hexagonal patterning (Figure 1C) similar to what can be observed in the cuticle of extant beetles, whereas the internal surface is comparatively smooth (Figure 1D,E). In the cross section, the cuticle appears largely amorphous, although the outermost portion displays distinct layering (Figure 1F). When visualized under TEM, the broadly homogenous internal texture is readily apparent (Figure 1G). Notably though, the elytra exhibit an outer lamellar structure with a total thickness of about 500 nm that consists of at least four electron-dense layers separated by thinner, more electron-lucent bands (Figure 1H). In addition, two different electron-lucent features were observed under TEM: long, vertical rifts (Figure 1G,H) and submicron fibrillary structures present predominantly in the ventral (lower) part of the section (Figure 1I–K). Setae- and/or sensilla-like bristles (Figure 1L), and features that may be related to the locking system that attaches the elytra to the thorax (see, e.g., [50,51]) were also evident under SEM (Figure 1M,N). 

Associated with the inferred cuticular remains are two different sedimentary microfabrics that display impressions from the fossil. One fabric appears dense with a regular patterning, whereas the other one has less distinct hexagons, appears more granulate (occasionally only as rounded crystals) and is significantly less coherent (Figure 1O). 

**Figure 2:**
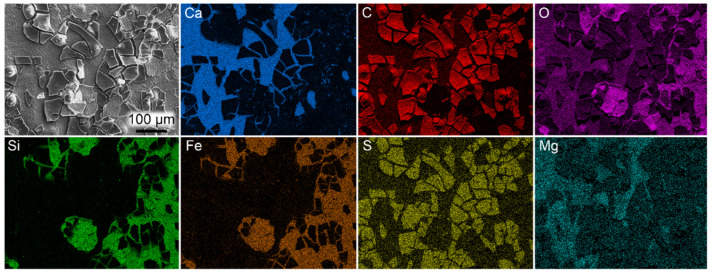
SEM-EDX elemental maps obtained from the fossil beetle elytra (FUM-N-17627). C, carbon (**red**); Ca, calcium (**blue**); Fe, iron (**orange**); Mg, magnesium (**cyan**); O, oxygen (**purple**); S, sulfur (**yellow**); Si, silicon (**green**).

EDX analysis revealed enrichment of carbon and sulfur in the beetle remains relative to the surrounding sediment (Figure 2). Other investigated elements (e.g., Si, Fe and Ca) are preferentially concentrated to the sediment. The two types of microfabrics that were observed in close association with the elytral fragments differ in composition (Figure 2). The dense mineral is enriched in calcium and oxygen (likely representing the enclosing calcitic concretion), whereas the granulate phase is dominated by silicon, iron and oxygen. 

**Figure 3:**
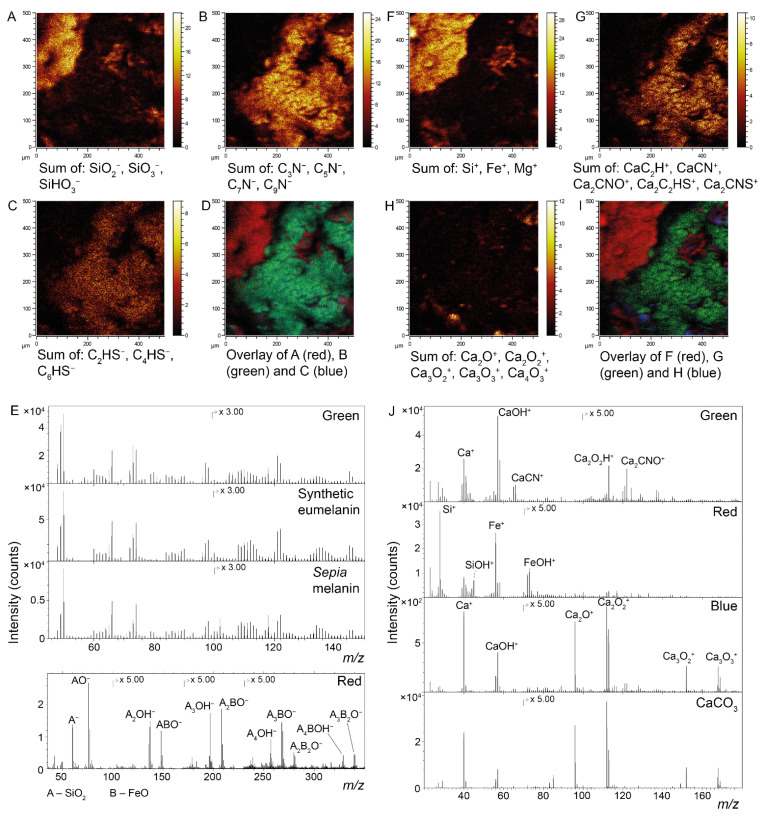
ToF-SIMS characterization of fossil beetle elytra (FUM-N-17627). (**A**–**D**) Negative ion images representing (**A**) silica, (**B**) eumelanin and (**C**) organic sulfur, together with (**D**) an overlay image in which silica is depicted in red, eumelanin in green and organic sulfur in blue. (**E**) Negative ion spectra obtained from selected ROIs that correspond to the green (eumelanin) and red (silica) areas in (**D**), respectively, where the spectrum from the green area is compared against reference spectra of synthetic and natural eumelanin standards. The spectrum from the red area demonstrates the generation of mixed cluster ions of silica and iron oxide. (**F**–**I**) Positive ion images representing (**F**) the mixed silica/iron oxide phase, (**G**) Ca-containing organic complexes and (**H**) calcite, together with (**I**) an overlay image in which silica/iron oxide is depicted in red and the Ca-containing organics are green and calcite is in blue. (**J**) Positive ion spectra from the red, green and blue areas in (**I**). The spectrum from the blue area is compared against a reference spectrum of calcite.

ToF-SIMS data obtained from the surface of the dark matter provided molecular evidence for the presence of the pigment eumelanin (Figure 3). Melanin identification was conducted by comparisons of negative ion spectra acquired specifically from dark-matter areas with reference spectra obtained from synthetic and natural variants of eumelanin. This procedure demonstrated a detailed spectral agreement both with regards to exact *m/z* values and the relative intensity distribution of all major peaks associated with the eumelanin molecular structure [29,34,52]. Furthermore, spectral comparisons of the fossils and eumelanin standards by PCA that included all eumelanin-related ions indicated peak intensity distributions of the beetle that are consistent with eumelanin standards (Figure S1). Sulfur-containing organic ions were also associated with the dark matter of the fossil (Figure 3C). Negative ion images further showed the presence of a material that displayed high signal intensity from silica-related ions. Mass spectra extracted from theseareas revealed a series of peaks corresponding to mixed (FeO)_m_(SiO_2_)_n_ cluster ions, indicating a mixed silicon/iron mineral phase (Figure 3E). Positive ion ToF-SIMS data confirmed the presence of a mixed silicon/iron mineral phase and, furthermore, identified calcite as the calcium-rich mineral (through detailed spectral agreement with a calcium carbonate standard; Figure 3J). Positive ion data further showed the presence of calcium in the form of Ca-containing organic fragment ions on the dark matter surface of the fossil (Figure 3G), although calcium was not observed during our (less sensitive) EDX analyses. 

### 4.2. FUM-N-11263 and FUM-N-10904

**Figure 4:**
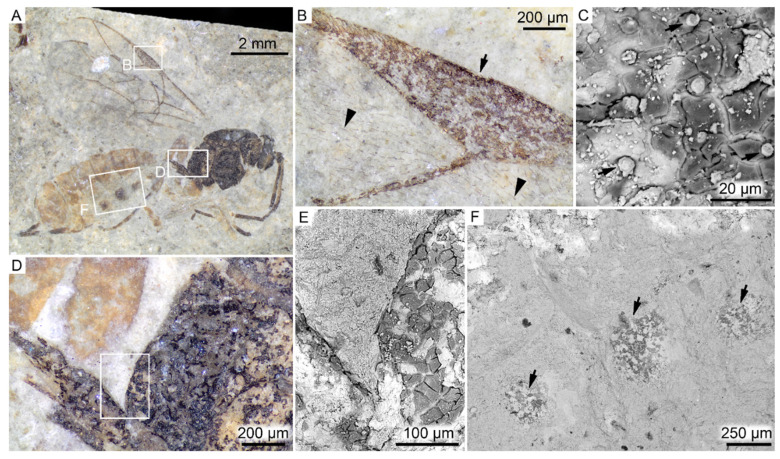
FUM-N-11263 (Hymenoptera). (**A**) Optical microscopy image of the fossil wasp with a dark thorax and head, and yellow-tinted abdomen and legs displayed in lateral view. (**B**) Higher magnification image showing brown pterostigma (arrow), a wing vein and the wing surface with abundant microtrichia (arrowheads). (**C**) SEM micrograph of inferred cuticular fragments in the pterostigma together with broken bristles (arrows). (**D**) Optical microscopy image of the narrow section joining the thorax and abdomen, displaying differences in preservation between the dark-colored matter and yellow-colored legs. (**E**) SEM micrograph of the area demarcated in (**D**) showing inferred cuticular remains. (**F**) SEM micrograph of the ventral side of the abdomen with carbonaceous spots (arrows) presumably representing relict cuticle.

**Figure 5:**
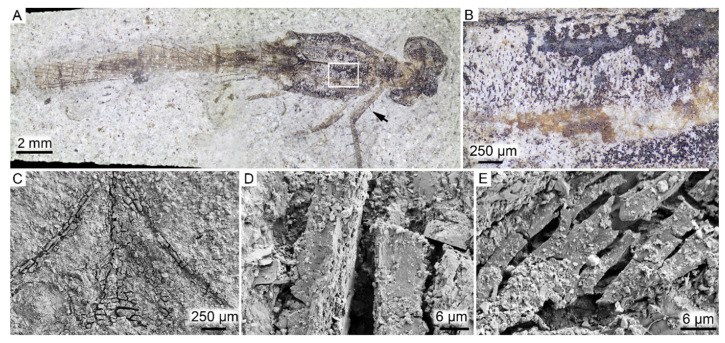
FUM-N-10904 (Odonata). (**A**) The fossil damselfly displayed in oblique dorsal view under conventional light. Note folded wings that rest on the abdomen, and apparent color patterns (e.g., pale shoulder stripes) on the legs and thorax. Arrow indicates location of Figure S3. (**B**) Close-up view of the area demarcated in (**A**) (upper part of the thorax) displaying black- to brown-colored matter. The latter is localized to the shoulder stripe. (**C**) SEM micrograph showing those microstructures that produce the outline of the fossil. (**D**) Higher magnification SEM image of block-like cuticular vestiges. (**E**) SEM micrograph of inferred cuticular residues in one of the legs.

Both FUM-N-11263 (Hymenoptera; Figure 4) and FUM-N-10904 (Odonata; Figure 5) are preserved as flattened yet fully articulated specimens. Under light microscopy, no obvious internal structures were evident. Instead, the remains visible on the bedding planes are interpreted as being solely cuticular in origin. In the wasp, cuticle fragments are present only in parts of the fossil that are visibly dark-colored (i.e., the head, thorax, wing veins and parts of abdomen). In addition, microtrichia—minute hair-like cuticular protuberances with various functions [53]—can be seen covering nearly the entire wing surface (Figure 4B). In the damselfly, fossilized the remains display color patterns and variations in hue, ranging from black to brown (Figure 5A,B). 

**Figure 6:**
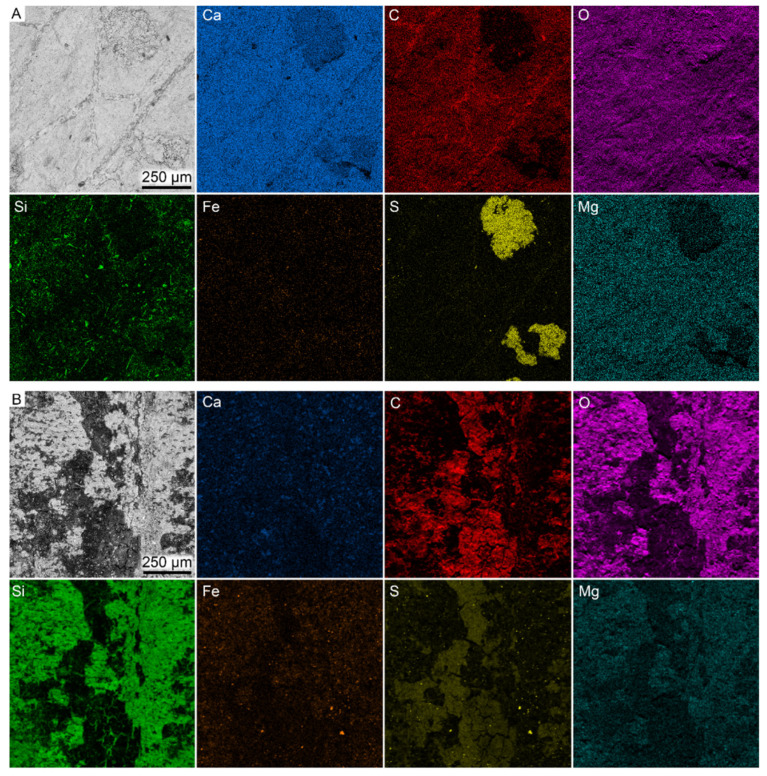
SEM-EDX elemental maps obtained from (**A**) wing veins in the wasp, FUM-N-11263, and (**B**) thorax of the damselfly, FUM-N-10904. C, carbon (red); Ca, calcium (blue); Fe, iron (orange); Mg, magnesium (cyan); O, oxygen (purple); S, sulfur (yellow); Si, silicon (green).

Elemental data from the two specimens (Figure 6) showed that only carbon and sulfur were concentrated to the fossil remains, although this enrichment was rather weak in the wasp. Trace elements, such as iron, occurred in low concentrations and were primarily associated with the sediment. 

**Figure 7:**
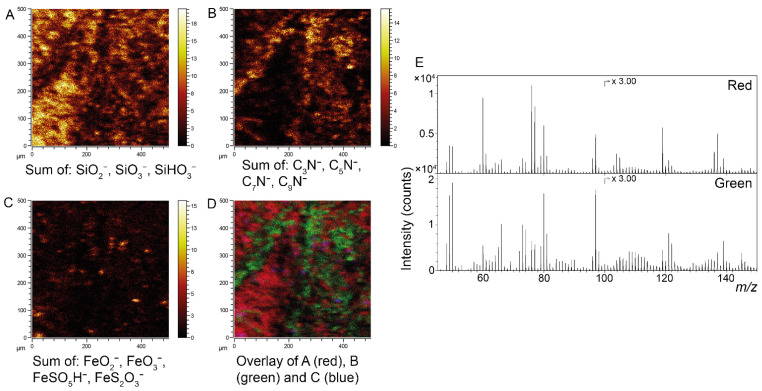
ToF-SIMS characterization of the fossil damselfly (FUM-N-10904). (**A**–**D**) Negative ion images representing (**A**) silica, (**B**) eumelanin and (**C**) iron oxide/sulfate, together with (**D**) an overlay image in which silica is presented in red, eumelanin in green and iron oxide/sulfate in blue. (**E**) Negative ion spectra from ROIs representing areas with high signal intensity from sediment-related ions (red; top) and eumelanin-associated ions (green; bottom), respectively.

Similarly to the beetle described above, the surfaces of the damselfly residues showed strong spectral agreement with reference spectra of both synthetic and natural eumelanin standards (Figure 7). In the wasp, however, a number of key nitrogen-bearing ions (at *m/z* 50, 74, 98, 122 and 146) showed considerably weaker signal intensities compared to the eumelanin standards, rendering confident molecular determination difficult. Spectral comparisons of the fossils and eumelanin standards by PCA showed peak intensity distributions in the damselfly that were consistent with eumelanin standards, whereas they clearly deviated in the wasp (Figure S1). Additionally, there were only minor differences between the dark and yellow areas of the wasp and adjacent (inorganic) matrix (Figure S2). In the damselfly, the sediment is composed mainly of silicate minerals with additional particulate structures of iron oxide/sulfate (Figure 6B and Figure 7), possibly representing former pyrite framboids. The association between eumelanin and cuticular residues, as well as iron oxide/sulfate and remnant framboids, was further demonstrated by superimposing SEM and ToF-SIMS images of seta on the front leg of the damselfly (Figure S3; location indicated in Figure 5A). 

## 5. Discussion

Insects are generally considered as “soft” organisms that lack naturally biomineralized body parts [54]. However, exceptional preservational conditions occasionally ensure the long-term survival of these otherwise labile animal remains. Decay-prone tissues, e.g., musculature and internal organs, generally require early diagenetic mineral formation to be incorporated in the fossil record [55,56]. Such mineral replacements can result in a high degree of morphological fidelity that include retained three-dimensionality (e.g., [14]). A variety of authigenic minerals are known to be involved in the fossilization of insect carcasses, including pyrite [19,57], calcium carbonate [9], calcium phosphate [58,59] and silica [60]. Alternatively, when authigenic mineralization does not occur, labile tissues can be preserved as organic remains (e.g., [11,15,61,62]).

Although not biomineralized, insect cuticle is a relatively rigid material, something that is attributed to sclerotization—a process in which the exoskeleton is hardened by means of covalent crosslinking between protein molecules [54]. Our microscopic investigation revealed that the insect residues examined herein are exclusively cuticular in origin, indicating that decay progressed until only such comparatively degradation-resistant body parts remained. Our elemental data further showed a concentration of carbon and, to a lesser extent, sulfur, associated with the fossil remnants, indicating that they are predominantly organically preserved. The elevated sulfur levels may indicate diagenetic incorporation of environmental sulfur into the eumelanin molecular structure, a process that has been suggested to enhance the recalcitrance and preservation potential of organic materials ([63] and references therein). As indicated by previously published carbon isotope compositions [47], the sea floor environment facilitated anaerobic microbial decomposition, probably via sulfate reduction [47]. The sulfur produced during this process could have contributed to the precipitation of pyrite [47]. However, although bacterial activity is commonly inferred to be associated with pyritization, fossils preserved in pyrite have yet to be reported from the Stolleklint Clay and Fur Formation to suggest that the conditions for widespread pyritization were not fulfilled in these deposits. Nonetheless, bacterial biofilms have been postulated to have aided the preservation of Fur Formation insects via protection from disintegration while the carcasses were sinking to the bottom [21,22]. Notably though, we did not find any evidence for microbial biofilms in our material.

The bulk of the organic matter in insect exoskeletons comprises a cross-linked chitin-protein complex [54]. Traces of this complex have been reported mainly from comparatively young fossils ([64,65,66,67] but see also [68]). In geologically older samples, original components are thought to have transformed into more stable (poly)aromatic and aliphatic compounds [61,69,70,71,72]. However, such geopolymers could not be reliably identified in our fossil samples. Instead, our Eocene insects appear to consist predominantly of eumelanin (or breakdown derivatives thereof). ToF-SIMS analyses of the beetle and damselfly provided strong evidence for the presence of preserved eumelanin and, in addition, indicate that this pigment constitutes a major fraction of the organic material in the cuticular residues. In contrast, eumelanin could not be confidently identified in the wasp due the divergent intensity distribution of nitrogen-bearing ions, which suggests further breakdown of the eumelanin biomacromolecule. Melanins in the cuticle of insects not only contribute to visual effects (color patterns) but also have other roles, including immunological defence and UV-protection [73]. Furthermore, cuticle melanogenesis is also intimately linked to the sclerotization process [74,75]. A tyrosine-mediated pathway is responsible for the production of melanins in the cuticle, but when certain dopamine-derived intermediates undergo crosslinking reactions with proteins, the tissue instead hardens (i.e., becomes sclerotized [76]). In most cases, pigments are incorporated into the exoskeleton through this process [75,76]. Despite a growing scientific interest in biochromes and colors of ancient organisms, few studies have hitherto been directed towards pigment residues in fossil insects (we are aware of only three publications in which an original biomolecule has been chemically identified [5,77,78]).

It cannot be completely excluded that remains of other organic components are also present in the fossil. For example, whereas eumelanin is preserved in a state that it is identifiable by ToF-SIMS, other components (chitin, protein and other pigments) could have been broken down into a heterogenous mixture of degradation products (e.g., aliphatic and aromatic hydrocarbons), each with a low concentration that could make it difficult to identify. Furthermore, cuticular components such as chitin would most likely overlap spatially with eumelanin in the sample, thereby preventing the possibility to extract spectra specifically from these other components and, consequently, aggravating their identification. 

Interestingly, in all three insects, body parts that likely were originally heavily melanized appear to better preserve cuticular remains. For instance, in the wasp, cuticle fragments were detected only in dark areas of the fossil (i.e., head, thorax and wing veins, as well as in smaller spots on the abdomen). Conversely, the abdomen and legs (where such fragments are not observed) were likely dominated by yellow-colored pigments and, thus, may have lacked substantial eumelanin deposits [54,79]. Similarly, organic structures were not observed to the same extent in the pale shoulder stripes of the damselfly, and the elytra additionally seem to constitute the thick, heavily pigmented dorsal portion only (our imaging analyses did not recover any evidence for the thinner ventral membranous part, hemolymph cavities or trabeculae). These observations not only suggest that eumelanin readily preserves, but also that it may constitute the bulk of the fossil remains or, alternatively, that it provides properties to the fossilized tissues that facilitate their preservation. Accordingly, we hypothesize that the lack of preserved cuticular remains in some areas of the fossils was due to limited initial eumelanin deposits. 

In recent years, chemical evidence of eumelanin has been documented in a broad range of animal fossils from the laminated clay of the Stolleklint Clay and in calcareous concretions of the Fur Formation [5,29,30,31,33,34]. The precise processes responsible for such a widespread presence are, however, not yet fully understood. Still, the high preservation potential of the biochrome is often attributed to its unique molecular structure. Experimental data indicate that it resists enzymatic and chemical degradation mechanisms [80,81,82,83], and enhances the strength of tissues and resistance to bacterial decay [84,85,86]. This may be a consequence of its molecular bond arrangement that has the ability to absorb optical and chemical energy and dissipate it as heat throughout the entire molecular structure, which grants protection against UV-light and suppresses free radicals [29,87,88]. Such factors may contribute to the retention of eumelanin in the fossil record by stabilizing it against decay and providing an inherent resistance to diagenetic alteration [89]. Moreover, the relatively mild geothermal conditions of the Stolleklint Clay and Fur Formation [90,91] presumably limited breakdown (previous studies have indicated that elevated burial temperature is a major factor controlling the preservation of eumelanin [92] as well as other molecular components and structures in arthropods [11,61,66,69]). 

The fossil beetle displays a higher degree of structural fidelity than the two other fossils. The rigid cuticle of extant beetles contains internal lamination and multiple pore canals [74]. In our specimen, however, the relict cuticle largely lacks internal laminae and instead appears to be more-or-less amorphous. A similar condition has previously been documented in both experimentally matured cuticles [69] and some fossil beetles [11] and probably reflects degradation and alteration of the chitin–protein complex during diagenesis [69]. Nevertheless, some conspicuous ultrastructural features were still observed in the cuticular remains. The electron-lucent, subvertical structures closely resemble pore canals (see [11], Figure 7C), and the submicron-scale fibril-like features (which, to our knowledge, are previously undocumented in fossil insects) may represent remnant pore canal filaments and/or chitin microfibrils (see [93], Figure 4E). Most notably, however, the cuticle displayed a number of distinct epicuticular layers that correspond to multilayer reflectors that create structural colors in extant insects. Structural colors have a long evolutionary history [10,94,95] and are known to have roles in, e.g., camouflage, mating and visual communication [96,97,98]. The photonic structures that generate these colors vary extensively in morphology, but the most extensively studied mechanism is the multilayer reflector. These reflectors consist of alternating layers with high and low refractive indices that collectively interact with light [99] and are the most common features that produce structural colors in modern beetles (often in the form of metallic shine or iridescence [97]. The multilayered structures can, for instance, be generated by stratified deposition of pigments, such as melanins or pteridines, and chitin [95,100,101]. In rare cases, such delicate structures are preserved also in fossil insects [10,11,102,103,104] and potentially can reveal aspects of the original colors and their functions in these ancient animals [10,104]. Notably, all previous reports of fossilized structural coloration in insects are from lacustrine deposits or amber [10,11,102,103,104], making this the first occurrence of preserved reflectors in a fossil insect preserved in a marine setting. In addition to structural colors, the elytra also provided molecular evidence of eumelanin. To the best of our knowledge, FUM-N-17627 represents the first fossil in which both structural colors and chemical evidence of a pigment have been documented. 

Associated with the beetle remains were calcium carbonate-dominated inorganics, representing the entombing concretion, as well as a silicon- and iron-rich mineral. We interpret the former as having formed early during diagenesis and been in close contact with the fossil based on the pristine appearance of the hexagonal cuticular impressions. This interpretation is further supported by our ToF-SIMS data, which show Ca-containing organic fragment ions localized to the dark-colored fossil matter, indicating a calcium-rich coating. Notably, calcium was not detected during our EDX analyses, suggesting that this element is present only at the surface of the dark matter (considering the high surface sensitivity of ToF-SIMS relative to EDX). Calcareous concretions are often considered to have sheltered newly formed fossils from both dissolution and compressional effects [105,106]. Indeed, insect fossils of mo-clay deposits have been previously noted to be especially well-preserved in concretions [1]; however, currently, further research is needed to better understand what role rapid calcium carbonate encapsulation played in the fossilization process. The formation of a silicon- and iron-rich mineral phase is evidently not associated with all insects and, thus, might represent a temporary (and local) event of diatom breakdown and/or an increased concentration of dissolved iron compounds, potentially from nearby volcanic eruptions [56]. 

## 6. Conclusions

To gain a better understanding of the retention of insect body fossils in the rock record, we investigated three exceptionally preserved specimens from the lowermost Eocene Stolleklint Clay and Fur Formation of Denmark. Our analyses show that these fossils are all preserved as organic but largely compressed remains of the exoskeleton. Specifically, ToF-SIMS data obtained directly from the cuticle revealed clear evidence of the natural pigment eumelanin, which seemingly dominates the dark-colored residues. Moreover, the beetle elytra exhibit high morphological fidelity with several unique nanostructures. Notably, TEM revealed remnants of an epicuticular multilayer reflector, a biophotonic structure that produces structural colors in modern insects. Our fossils further indicate a potential preservational bias: the preservation potential seems to be greatly diminished in regions of the cuticle that lacked substantial eumelanin deposits. Eumelanin has an inherent resistance to decay and, thus, may remain when most other components have degraded or been lost during diagenesis. The results of our study provide novel insights into the taphonomy of insect assemblages preserved in marine paleoenvironments.

## Data Availability

All data generated by this study are available in this manuscript and the accompanying Supplementary Materials.

## Supplementary Materials

The following supporting information can be downloaded at: https://www.mdpi.com/article/10.3390/biology11030395/s1. Figure S1: PCA analysis; Figure S2: ToF-SIMS spectra of FUM-M-11263; Figure S3: SEM and ToF-SIMS images from FUM-N-10904.
