# Supplementary material for: Preservation and Taphonomy of Fossil Insects from the Earliest Eocene of Denmark
Source: Biology (Basel). 2022 Mar 3;11(3):395. doi: 10.3390/biology11030395 (PMC8945194; doi:10.3390/biology11030395)
Supplement: Supplementary file 1 [file biology-11-00395-s001.zip › biology-1603692-supplementary.pdf]

Supplementary Materials

Preservation and taphonomy of fossil insects from the earliest Eocene of Denmark

Miriam Heingård, Peter Sjövall,
Bo P. Schultz, René L. Sylvestersen
and Johan Lindgren

Figure S1

PCA analysis comparing eumelanin-related ions of three insect fossils (FUM-N-11263, FUM-N-10904 and FUM-N-17627) and two eumelanin standards.

Figure S2

Positive and negative ToF-SIMS spectra of FUM-N-11263.

Figure S3

SEM and ToF-SIMS images from FUM-N-10904, showing the association of eumelanin to cuticle residues and iron oxide/sulfate to framboid structures.

Figure S1

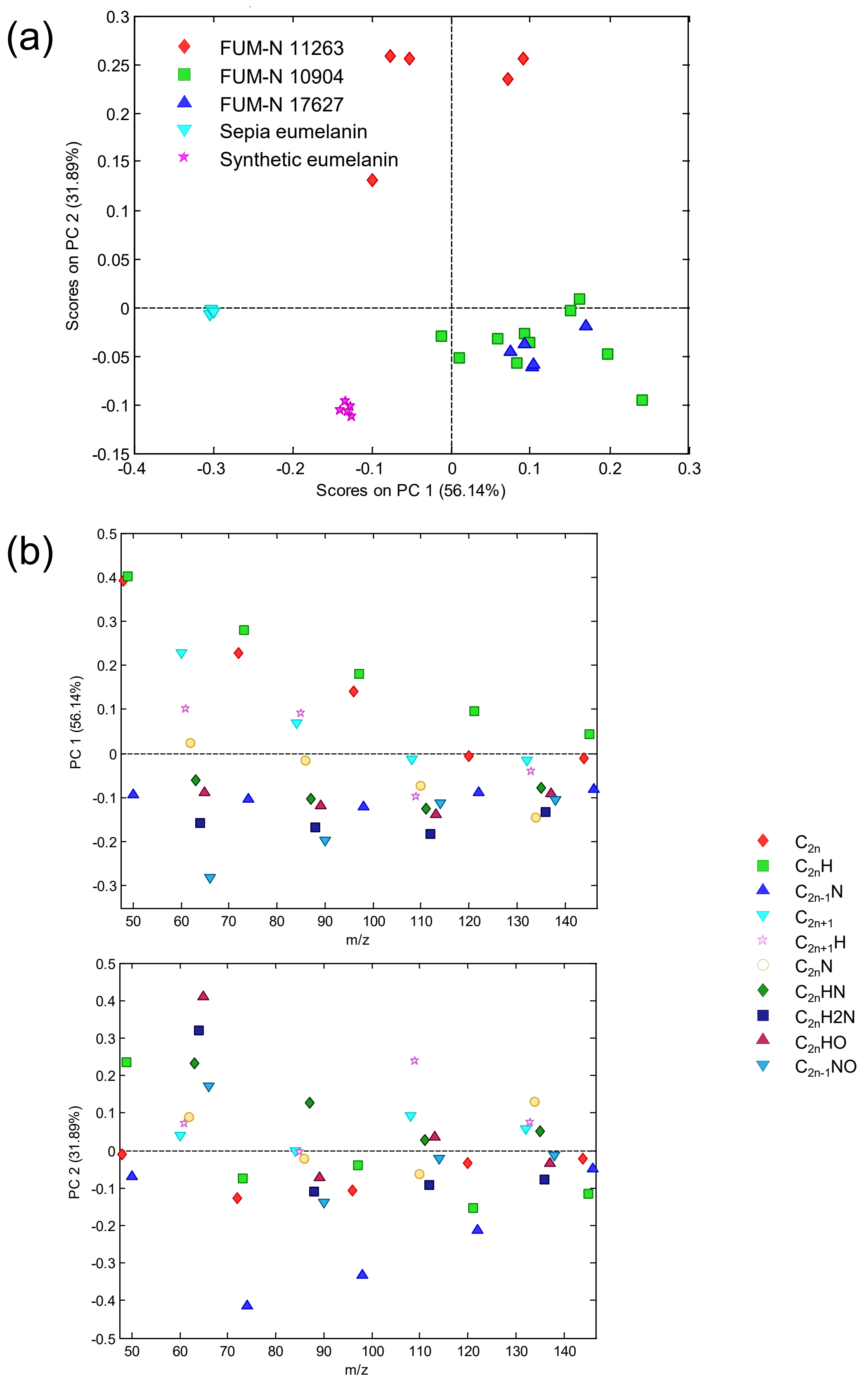

(a) Score plot of PC2 versus PC1. (b) Loadings of PC1 (top) and PC2 (bottom) for secondary ions of different categories.

Figure S2

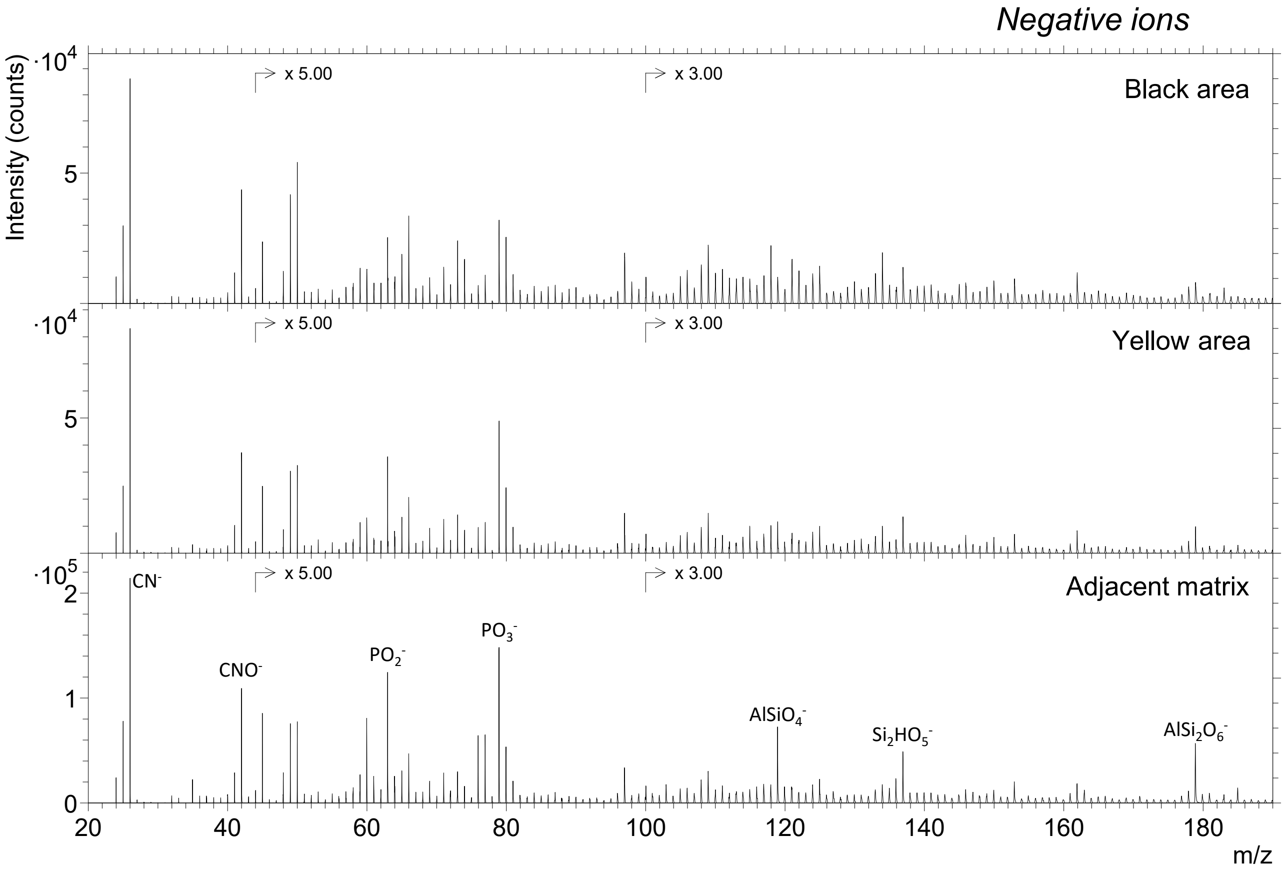

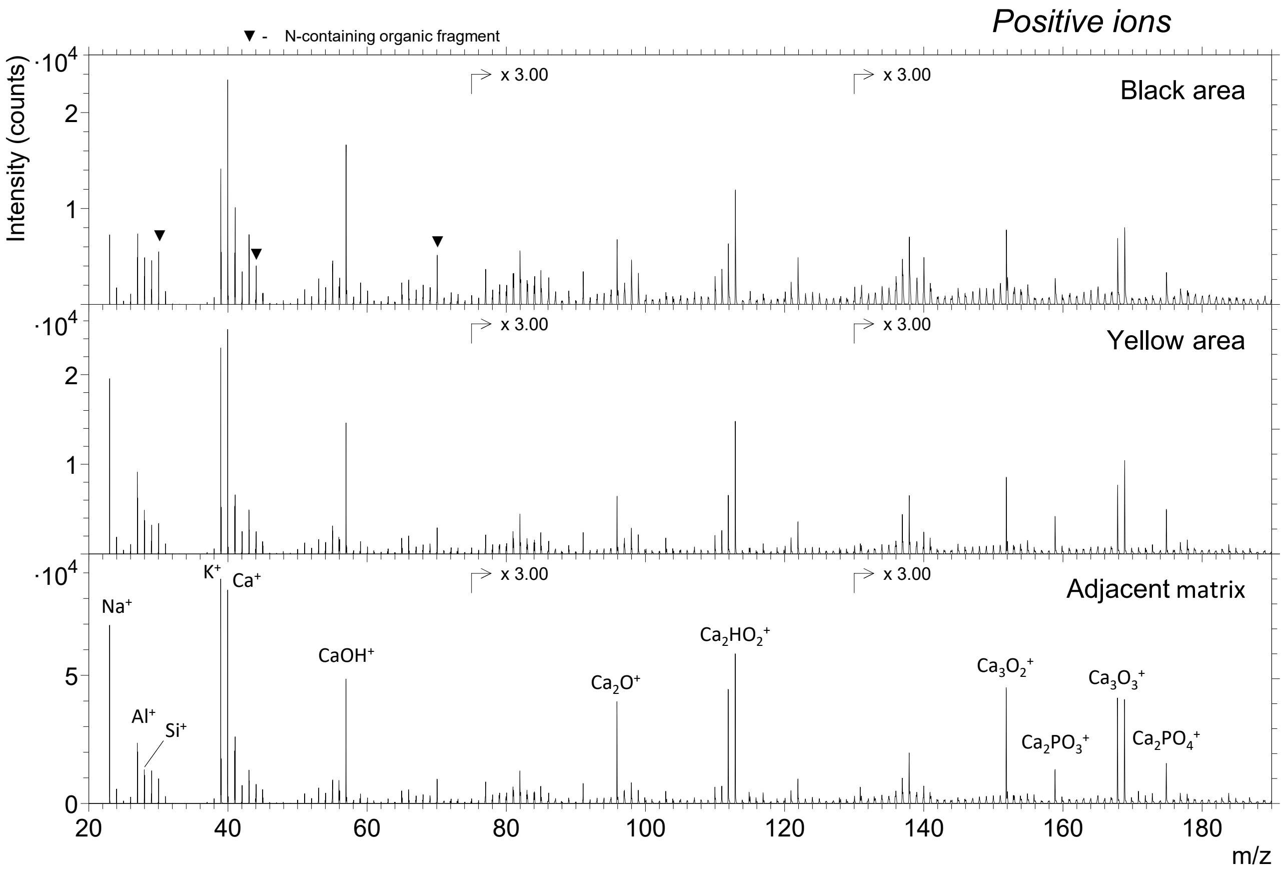

Black and yellow areas refer to fossil regions in the light microscopy image (Figure 4A).

Figure S3

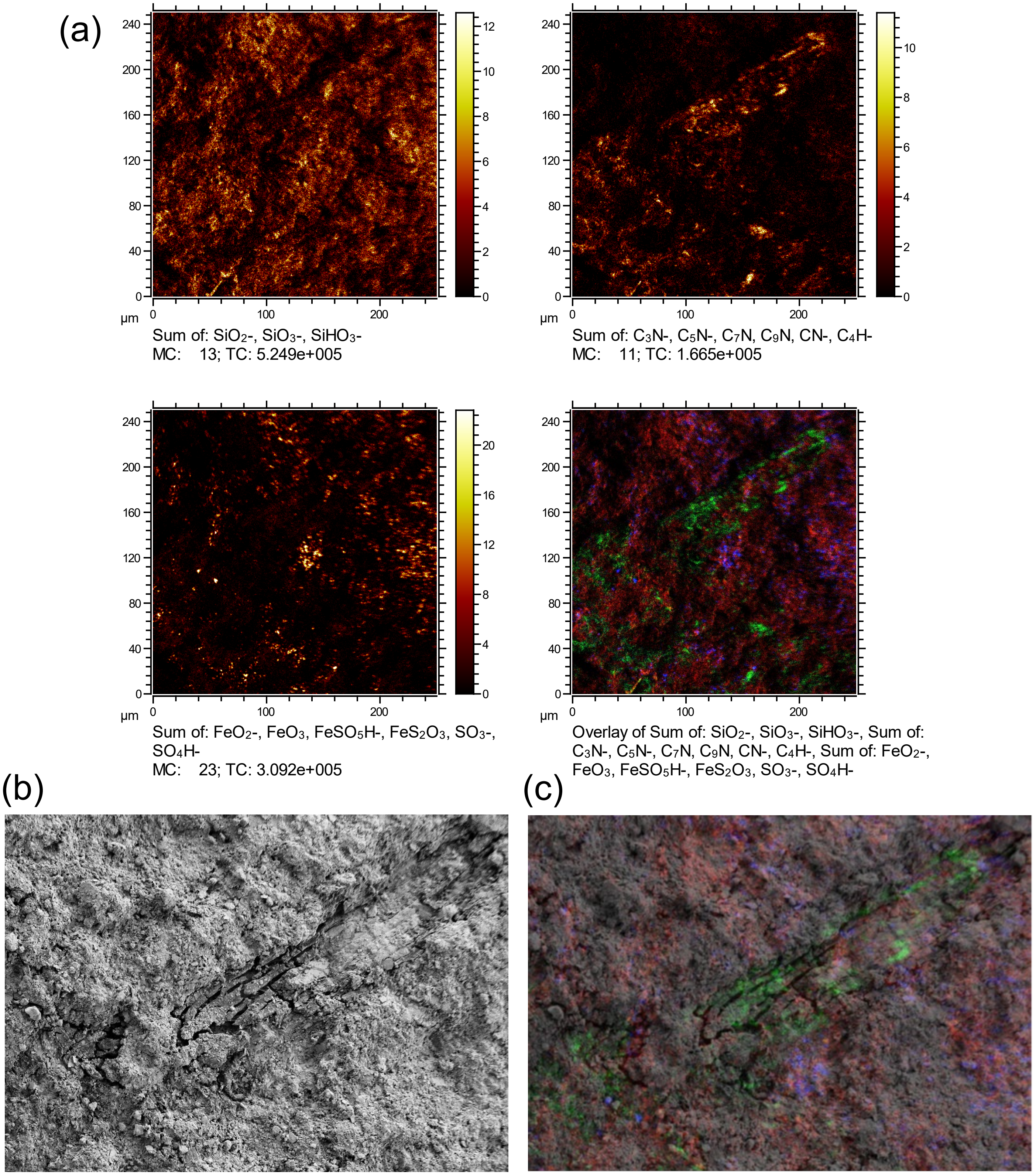

(a) TOF-SIMS images representing silica, eumelanin, iron oxide/sulfate and an overlay image of these three. (b) SEM micrograph of the same area as in (a), and (c) overlay of SEM micrograph in (b) and overlay TOF-SIMS image in (a). The images show protruding seta on the front leg of the fossil damselfly (arrow in Figure 5A).
